# Genome-Wide Identification, Evolutionary Analysis, and Functional Studies of *APX* Genes in Melon (*Cucuis melo* L.)

**DOI:** 10.3390/ijms242417571

**Published:** 2023-12-17

**Authors:** Jiayan Song, Zicheng Zhu, Taifeng Zhang, Xiaobing Meng, Wencheng Zhang, Peng Gao

**Affiliations:** College of Horticulture and Landscape Architecture, Northeast Agricultural University, Harbin 150030, China; songjiayan0310@163.com (J.S.); zzc1983sc@163.com (Z.Z.); ztf18104603353@163.com (T.Z.); 18703907080@163.com (X.M.); zwc18579109134@163.com (W.Z.)

**Keywords:** melon, salt stress, *APX* gene family, functional validation

## Abstract

The antioxidative enzyme ascorbate peroxidase (APX) exerts a critically important function through scavenging reactive oxygen species (ROS), alleviating oxidative damage in plants, and enhancing their tolerance to salinity. Here, we identified 28 *CmAPX* genes that display an uneven distribution pattern throughout the 12 chromosomes of the melon genome by carrying out a bioinformatics analysis. Phylogenetic analyses revealed that the *CmAPX* gene family comprised seven different clades, with each clade of genes exhibiting comparable motifs and structures. We cloned 28 *CmAPX* genes to infer their encoded protein sequences; we then compared these sequences with proteins encoded by rice APX proteins (OsAPX2), *Puccinellia tenuiflora* APX proteins (PutAPX) and with pea APX proteins. We found that the *CmAPX17*, *CmAPX24*, and *CmAPX27* genes in Clade I were closely related, and their structures were highly conserved. *CmAPX27* (*MELO3C020719.2.1*) was found to promote resistance to 150 mM NaCl salt stress, according to quantitative real-time fluorescence PCR. Transcriptome data revealed that *CmAPX27* was differentially expressed among tissues, and the observed differences in expression were significant. Virus-induced gene silencing of *CmAPX27* significantly decreased salinity tolerance, and *CmAPX27* exhibited differential expression in the leaf, stem, and root tissues of melon plants. This finding demonstrates that *CmAPX27* exerts a key function in melon’s tolerance to salt stress. Generally, *CmAPX27* could be a target in molecular breeding efforts aimed at improving the salt tolerance of melon; further studies of *CmAPX27* could unveil novel physiological mechanisms through which antioxidant enzymes mitigate the deleterious effects of ROS stress.

## 1. Introduction

Melon (*Cucumis melo* L.) has high nutritional value and is, therefore, a vegetable crop with notable economic significance [1,2,3]. Although melon has high water requirements, it is often grown in semi-arid areas; the use of saline water to irrigate melon in such regions results in reduced yield and quality [4,5]. Rises in global temperature, as well as the drought severity and frequency in melon production areas, have exacerbated the deleterious effects of salt stress on melon.

Soil salinization is thought to have had adverse effects on nearly one-fifth of global irrigated farmland [6]. Excessive soil salt concentrations can alter the ionic balance of plants, which can affect osmotic regulation and induce severe stress [7,8]. Exposure of plants to salt stress gives rise to excessive amounts of reactive oxygen species (ROS), including hydroxyl radicals, hydrogen peroxide, superoxide anion radicals, and singlet oxygen. This in turn leads to the destruction of important cellular components of plants, including lipids, organelles, proteins, and nucleic acids, eventually inducing apoptosis and the death of the plant [9,10,11]. The maintenance of ROS homeostasis in plants is thus critically important in high-salinity environments; ROS-scavenging activity is essential for inducing appropriate cellular signaling pathways and maintaining ROS homeostasis under such conditions.

The antioxidant system of plants maintains redox homeostasis when they face salt stress. The plant antioxidant enzymes that perform essential ROS-scavenging functions include catalase, superoxide dismutase, and ascorbate peroxidase (APX) [12,13]. Among them, APX (EC 1.11.1.11), which belongs to the peroxidase superfamily, utilizes ascorbic acid (AsA) as an electron donor to convert H_2_O_2_ into O_2_ and H_2_O through the ascorbate–glutathione pathway (ASC–GSH), thereby enhancing plants’ stress tolerance [14,15]. Thus, APX activity is significantly up-regulated after plants are exposed to environmental stress, including exposure to high salinity, high heavy metal concentrations, and drought [16].

*APX* genes are crucial for plants’ stress tolerance, and the number of *APX* genes varies among species. Previous studies have characterized 8 *APX* genes in *Arabidopsis thaliana* and rice [17,18,19]. In rice, *OsAPX2* overexpression could enhance the plant’s tolerance to drought, salt, and cold stress, whereas knockdown of the gene impairs the plant’s development and growth and hinders its responses to abiotic stress, indicating that *OsAPX2* is crucial for rice’s resistance to various types of abiotic stress [20]. In cotton, sorghum, wheat, *Populus trichocarpa*, tomato, kiwifruit, winter rapeseed, peanut, and watermelon, the numbers of *APX* genes identified so far are, respectively, 26, 9, 21, 11, 7, 13, 118, 166, and 5 [14,21,22,23,24,25,26,27,28]. Salt stress affects root growth and development in sweet potato; it has been observed that transgenic sweet potato plants with *APX* genes from pea exhibited significantly better salt stress tolerance and faster root growth and development than their wild-type counterparts [29]. In *Puccinellia tenuiflora*, overexpression of *PutAPX* enhances ROS scavenging and, thus, salt tolerance, while the effects of overexpressing *PutAPX* vary among organs; these observations collectively signify that *PutAPX* is a key gene conferring salt stress resistance in *Puccinellia tenuiflora* [30].

Here, through bioinformatics analysis, we identified 28 *CmAPX* genes in melon, which could be categorized into seven branches based on phylogenetic analysis. We analyzed the physicochemical properties, structure, and chromosomal locations of these genes; we also evaluated their phylogenetic relationships, analyzed patterns of gene collinearity, and compared their amino acid sequences and RNA-seq expression profiles. Based on these analyses, we predicted the following three candidate genes: *CmAPX17*, *CmAPX24*, and *CmAPX27*. We then analyzed the expression patterns of *CmAPX27*, which is a key gene that mediates salt stress resistance in melons, using transcriptomic and quantitative real-time polymerase chain reaction (qRT-PCR) assays, and confirmed its function using gene-silencing techniques.

## 2. Results

### 2.1. Identification of APX Genes in the Melon Genome

We identified 28 *CmAPX* genes in the genome of melon, which were named *CmAPX1*–*CmAPX28* (Table 1). Six *CmAPX* genes were on Chr11; five *CmAPX* genes were on Chr05; three *CmAPX* genes were on each Chr03, Chr07, and Chr12; two *CmAPX* genes were on Chr04; and the remaining chromosomes each had one *CmAPX* gene. Coding sequences (CDSs), introns, and exons of *CmAPX* genes were analyzed using the Cucurbitaceae database. The CDSs were 471 to 1251 bp (*CmAPX1/4*) in length and contained 1 (*CmAPX2/7*) to 12 (*CmAPX4*) exons; *CmAPX4* had the highest number of introns, and introns were lacking in *CmAPX2* and *CmAPX7*. The identified *CmAPX* genes were also subjected to protein sequence analysis using Expasy-Protparam. The results revealed that *CmAPX1*–*CmAPX28* encode proteins composed of 156 to 416 (*CmAPX1/4*) amino acids. These proteins exhibited an isoelectric point of 4.53 to 9.15, a molecular weight of 17.26 to 45.04 kilodaltons, and a GRAVY score of −0.411 (*CmAPX4*) to 0.04 (*CmAPX14*). CELLO v.2.5 was then employed to predict subcellular localization for these CmAPX proteins. A total of 13, 2, 5, and 8 proteins were predicted to be expressed in the extracellular matrix, plasma membrane, cytoplasm, and chloroplast, respectively.

### 2.2. Structure and Conserved Motif Analyses for the CmAPX Genes

**Figure 1:**
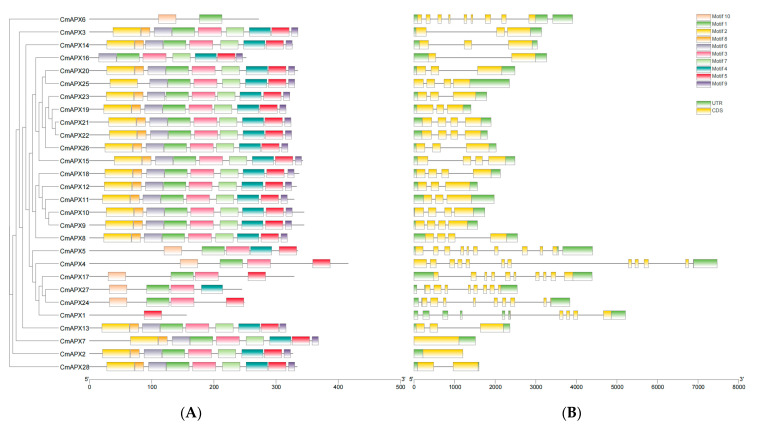

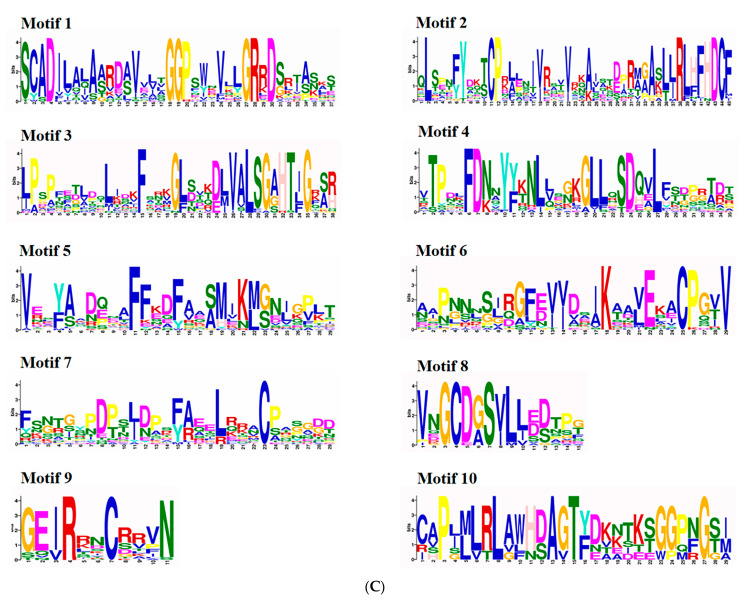
Conserved motif and structure analyses for the *CmAPX* genes. (**A**) Schematic diagram of the conserved motifs predicted in CmAPX proteins. Each conserved motif is shown in a specific color. The length of the amino acid sequence can be inferred using a ruler at the bottom. (**B**) Structure of *CmAPX* genes. (**C**) Length and amino acids of the 10 conserved motifs identified among the CmAPX proteins. Letter size corresponds to the frequency of the amino acid.

The conserved motifs and intron distribution of *CmAPX* genes were analyzed. Using the MEME software (http://meme-suite.org/, accessed on 20 January 2023), the amino acid sequences of the 28 CmAPX proteins were analyzed. We identified 10 conserved motifs among the 28 proteins, with the number of conserved motifs in each CmAPX protein ranging from 1 to 9 (Figure 1). *CmAPX3*, *CmAPX14*, *CmAPX20*, *CmAPX23*, *CmAPX19*, *CmAPX21*, *CmAPX22*, *CmAPX26*, *CmAPX15*, *CmAPX18*, *CmAPX12*, *CmAPX11*, *CmAPX10*, *CmAPX9*, *CmAPX8*, *CmAPX13*, *CmAPX7*, *CmAPX2*, and *CmAPX28* contained nine conserved motifs; *CmAPX25* contained eight conserved motifs; *CmAPX16* contained seven conserved motifs; *CmAPX5* contained five motifs; *CmAPX4*, *CmAPX17*, *CmAPX27*, and *CmAPX24* contained four motifs; and *CmAPX6* contained two motifs. *CmAPX1* encoded the shortest protein, which contained only one conserved motif. Overall, our findings suggested that the predicted conserved motifs might be crucial for the functions of CmAPX proteins.

There was a high degree of variation in the structure of *CmAPX* genes; *CmAPX4* had the most introns (11), and *CmAPX2* had no introns. *CmAPX4* was the longest, and *CmAPX2* was the shortest. The UTR region was absent at one end in four genes (*CmAPX25, CmAPX4*, *CmAPX7*, and *CmAPX2*).

### 2.3. Chromosomal Localization of CmAPX Genes and Collinearity Analysis of APX Genes

**Figure 2:**
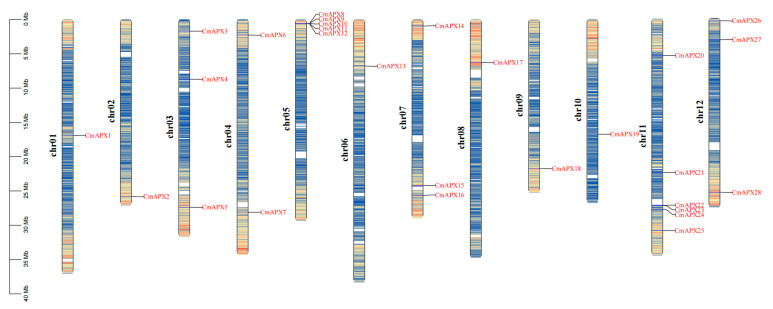
The distribution pattern of *CmAPX* genes on melon chromosomes.

**Figure 3:**
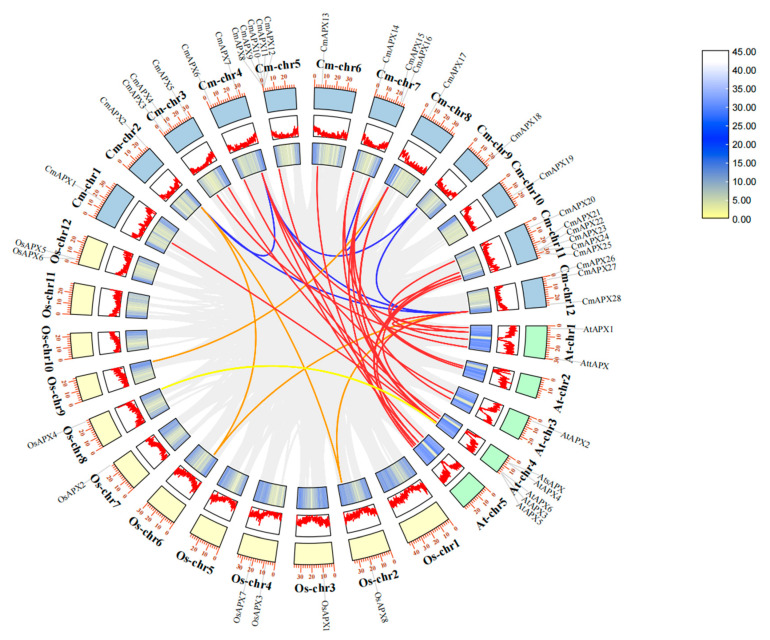
Collinearity analysis of *CmAPX* genes. Orange, red, and yellow lines denote sequence homology between the melon and rice, melon and Arabidopsis, and Arabidopsis and rice genomes, respectively; blue lines indicate sequence homology among melon chromosomes. The segmental duplication pairs among chromosomes are linked by lines.

The identified 28 *CmAPX* genes distributed on the 12 chromosomes of melon (Figure 2) and *CmAPX1*, *CmAPX2*, *CmAPX13*, *CmAPX17*, *CmAPX18*, and *CmAPX19* were located on 6 different chromosomes (Chr01, Chr02, Chr06, Chr08, Chr09, and Chr10). *CmAPX6* and *CmAPX7* were located on Chr04; Chr03, Chr07, and Chr12 each carried three *CmAPX* genes; and five *CmAPX* genes and six *CmAPX* genes were located on Chr05 and Chr11, respectively. We then conducted a collinearity analysis on the *APX* genes of three species of melon, *A. thaliana*, and rice, as well as within the three melon species, using the MCScanX function in TBtools software (v1.09867) to explore their evolutionary relationships (Figure 3). We detected 31 pairs of segmental duplications within 22 genes among three species. Collinearity was highest between *CmAPX* and *AtAPX* genes, and a total of 24 pairs were detected. Within melon species, *CmAPX7* was colinear with *CmAPX2* and *CmAPX28*. Other *CmAPX* genes were highly conserved.

### 2.4. Tissue Expression Profiles of CmAPX Genes

**Figure 4:**
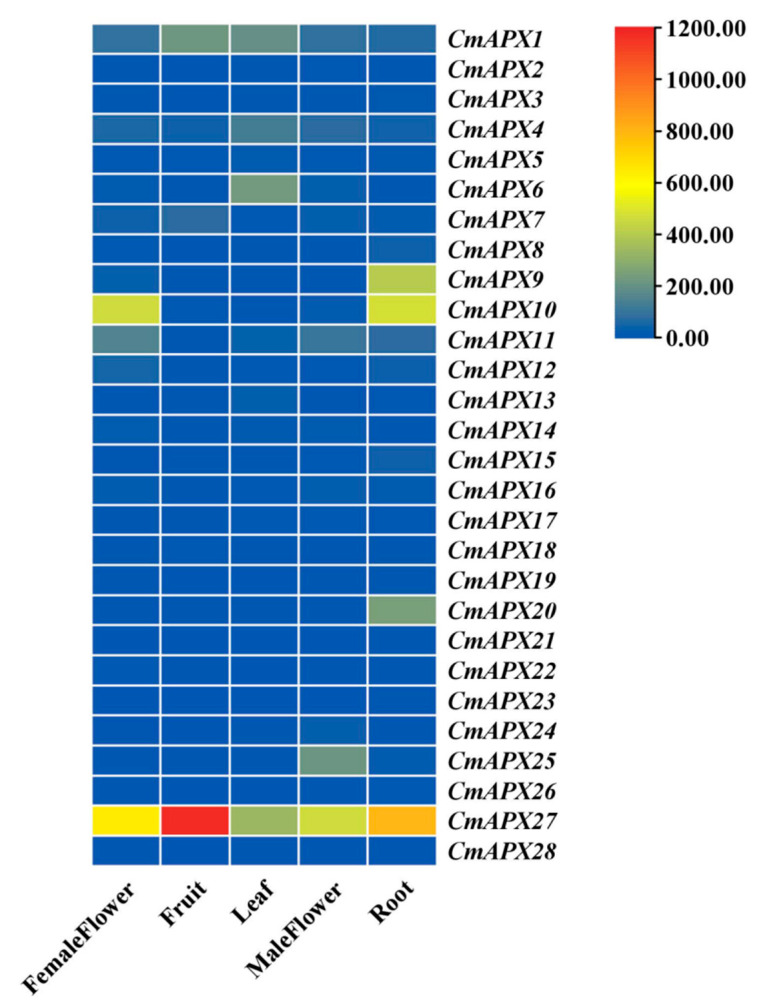
Tissue expression profiles of *CmAPX* genes.

The expression profiles of the 28 *CmAPX* genes were characterized in female flower, fruit, leaf, male flower, and root tissues of melon based on the tissue-specific RNA-seq data deposited in the Melon Genome Database. As shown in Figure 4, these genes were differentially expressed among the examined tissues. Notably, *CmAPX27* exhibited much higher expression levels in all the examined tissues, compared with other *CmAPX* genes, and distinctive tissue-specific expression patterns.

### 2.5. Phylogenetic Analysis and Gene Cloning

**Figure 5:**
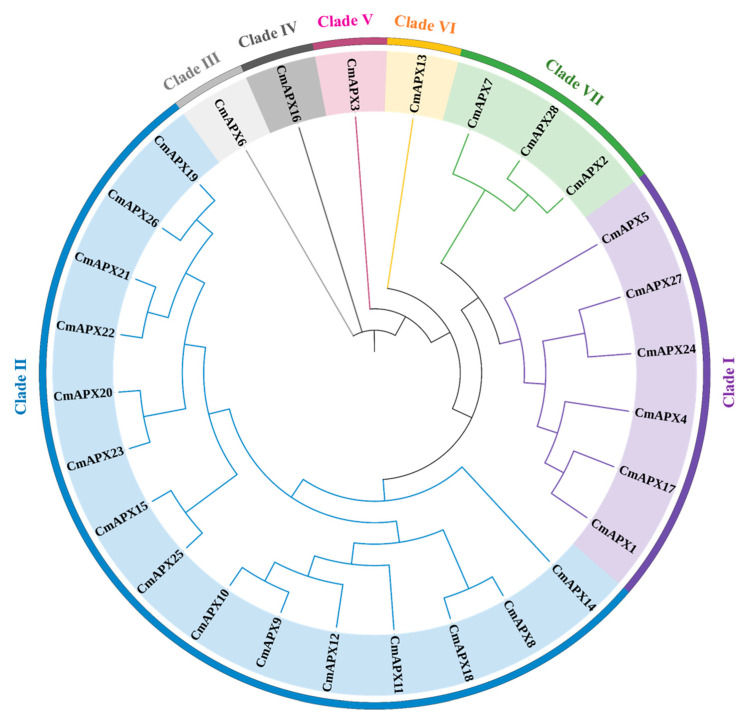
Schematic diagram of *CmAPX* genes in the phylogenetic tree.

To clarify the evolutionary relationships among the 28 *CmAPX* genes, we established a phylogenetic tree based on their encoded proteins (Figure 5). The 28 CmAPX proteins can be classified into seven clades. Clade I contained six *CmAPX* genes; Clade II contained 15 *CmAPX* genes; Clades III to VI each contained one *CmAPX* gene; and Clade VII contained three *CmAPX* genes. Genes belonging to the same clade displayed high similarity, which may indicate that they possess similar physiological functions.

**Figure 6:**
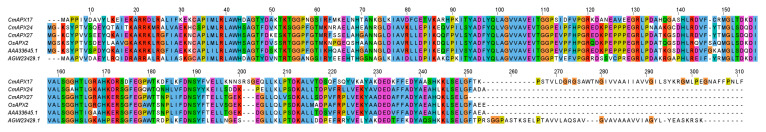
Amino acid sequence alignment diagram.

*OsAPX2* in rice, *APX* (*AAA33645.1*) in pea, and *PutAPX* (*AGW23429.1*) in *Puccinellia tenuiflora* are known to exert crucial functions in their hosts’ response to salt stress [21,30,31]. Therefore, we compared the deduced protein sequences of the identified 28 *CmAPX* genes with those encoded by *OsAPX2*, *AAA33645.1*, and *AGW23429.1* (Figure 6). We found that the sequences of CmAPX17, CmAPX24, and CmAPX27 were similar to those of OsAPX2, AAA33645.1, and AGW23429.1; the conservation in the structure of these genes suggests that these three *CmAPX* candidate genes are functionally similar to *OsAPX2*, *AAA33645.1*, and *AGW23429.1*. Thus, *CmAPX17*, *CmAPX24*, and *CmAPX27* in Clade I may be implicated with melon’s salt stress tolerance.

### 2.6. Expression Patterns of the Three Candidate Genes in the Context of Salt Stress

**Figure 7:**
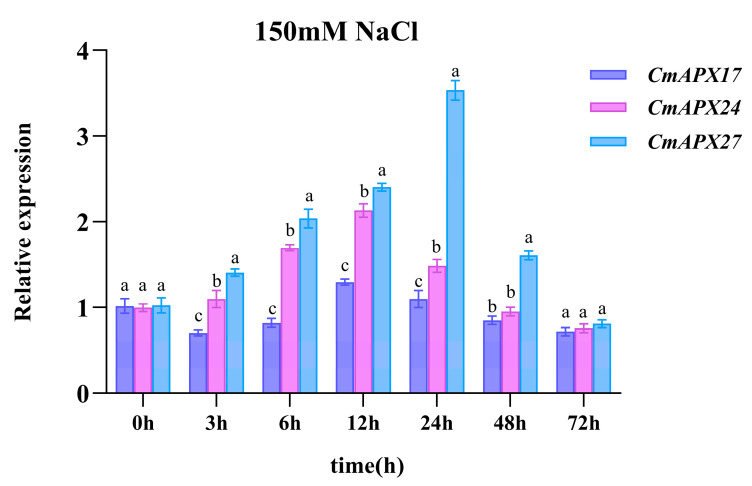
RT-qPCR analyses of the relative mRNA abundances of *CmAPX17*, *CmAPX24*, and *CmAPX27* genes in melon leaves after salt stress treatment for different periods (as indicated by the *x*-axis). Error bars indicate the standard error derived from three independent experiments. The lowercase letters denote that the differences among groups reach a statistically significant level of *p* < 0.05 in Duncan’s multiple range test.

Melon seedlings were subjected to salt stress treatment using NaCl (150 mM) for different periods (0 h, 3 h, 6 h, 12 h, 24 h, 48 h, and 72 h), after which the leaves were collected for gene expression analysis (Figure 7). Among the three candidate genes, *CmAPX27* exhibited the highest expression level at all time points examined, implying its crucial function in mediating melon’s response to salt stress.

### 2.7. Functional Validation of CmAPX27

**Figure 8:**
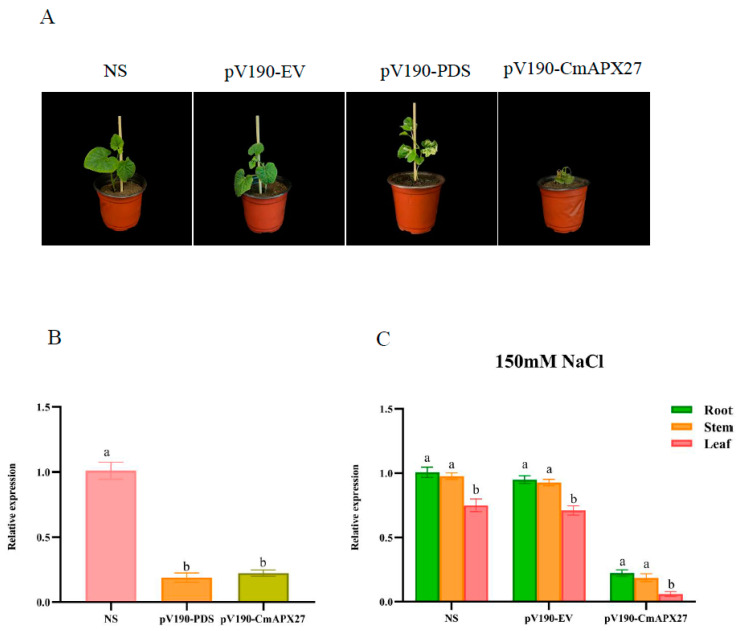
Phenotypes of melon plants after gene silencing and expression analysis. (**A**) The phenotypes of *CmAPX27*-silenced and non-silenced melon plants were determined following exposure to 150 mM NaCl stress in plants. NS, non-silenced control; pV190-EV, plants infected with the pV190 empty vector; pV190-PDS, plants infected with pV190-PDS; pV190-CmAPX27, plants infected with pV190-CmAPX27. (**B**) Analysis of pV190-PDS and pV190-CmAPX27 expression patterns after gene silencing. The lowercase letters denote that the differences among groups reach a statistically significant level of *p* < 0.05 in Duncan’s multiple range test. (**C**) Gene expression patterns of NS, pV190-EV, and pV190-CmAPX27 plants in rhizomes and leaves under 150 mM NaCl stress. The lowercase letters denote that the differences among groups reach a statistically significant level of *p* < 0.05 in Duncan’s multiple range test.

To determine the function of *CmAPX27*, we utilized the VIGS method to silence *CmAPX27* and *PDS* in the X055 variety. We also set up non-silenced (NS) controls and empty vector controls (plants infected with the pV190 empty vector, pV190-EV) for this experiment. Relative to the NS controls, the expression levels of *CmAPX27* and *PDS* were markedly down-regulated in both pV190-PDS and pV190-CmAPX27-infected plants (Figure 8B). pV190-EV, pV190-CmAPX27, and NS plants were subjected to 150 mM NaCl treatment; phenotypic observations were made, and the expression of *CmAPX27* in root, stem, and leaf tissues was determined (Figure 8A,C). The leaves of pV190-CmAPX27-infected plants were yellow, curled, and dry, and the expression of *CmAPX27* differed in leaf, root, and stem tissue after 48 h; *CmAPX27* exhibited higher expression in root and stem tissues than in leaf tissue. Seedlings died around 72 h. By contrast, leaf yellowing was only observed at the margin of leaves in NS plants and pV190-EV plants, and the leaves of these plants were only slightly curled. These findings indicated that *CmAPX27* is one of the main genes regulating melon’s salt stress response.

### 2.8. APX Activity Assay

**Figure 9:**
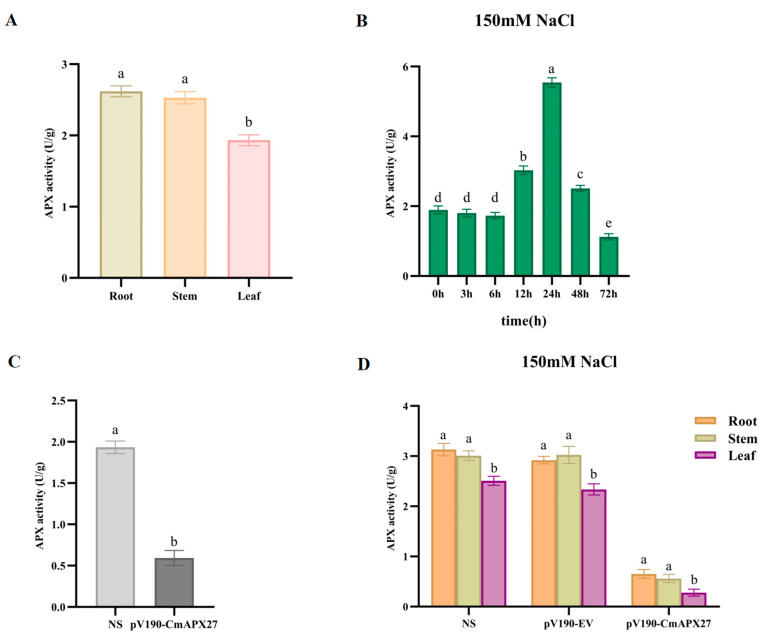
Variation in APX activity in melon plants. The lowercase letters denote significant differences in APX activity among groups (*p* < 0.05) according to Duncan’s multiple range test. (**A**) Determination of APX activity in melon tissues in the absence of salt stress. (**B**) APX activity of melon leaves at different times under 150 mM NaCl. (**C**) APX activity in leaves of NS plants and pV190-CmAPX27 plants in the absence of salt stress. (**D**) APX activity in various tissues of NS plants and pV190-CmAPX27 plants under 150 mM NaCl.

Assays of APX activity in the roots, stems, and leaves of melon revealed that APX activity was high in the roots and stems (Figure 9A). Under 150 mM NaCl, APX activity increased with the duration of stress exposure, but APX activity decreased at 48 h (Figure 9B). APX activity was significantly lower in pV190-CmAPX27 plants than in NS plants (Figure 9C). Under salt stress, APX activity was much lower in all tissues of pV190-CmAPX27 plants than in NS plants, and APX activity was greater in the roots and stems than in the leaves (Figure 9D).

## 3. Discussion

Saline soils lead to reductions in crop yields by disrupting biochemical and physiological events in plants [32]. Salt stress inhibits plant growth by inhibiting cell expansion [7,33]. Salt tolerance in melon is closely related to the amino acid content, hormone concentration, and the Na^+^/K^+^ ratio [34]. Excessive soil salt in melon production areas could cause ionic imbalances and hypertonic stress, which can negatively affect melon development and growth, eventually reducing the yield. Salt stress can induce ROS production and accumulation by triggering oxidative reactions in plants [35]. ROS can induce cell wall relaxation to affect cell expansion [7]. *APX* genes encode enzymes in the peroxidase family that can break down excess H_2_O_2_ in the cell, which prevents excessive ROS from destroying cells; thus, they play a key role in regulating the response to salt stress [36]. These genes are highly conserved because counterparts from several different species often encode the same conserved protein domain, and the sequence similarity of APX proteins from different species ranges from 93.6 to 96.8%. In this study, we identified a total of 28 *CmAPX* genes, each of which encoded an APX structural domain. Subcellular localization predictions revealed that melon CmAPX proteins were expressed in different locations compared with *A. thaliana* AtAPX and rice OsAPX proteins. The expression of CmAPX proteins at multiple sites suggests that these proteins are functionally diverse but all contribute to ROS scavenging in multiple organelles of plant cells.

The presence of *CmAPX* genes on all 12 chromosomes of melon provides further evidence for the high functional diversity of *CmAPX* genes. Analysis of the structure of *CmAPX* revealed that genes belonging to the same branch exhibited a similar distribution of exons, introns, and conserved motifs. The deduced protein sequences of CmAPX17, CmAPX24, and CmAPX27 were similar to those of OsAPX2, AAA33645.1, and AGW23429.1. This indicates that these genes are functionally similar and structurally conserved; thus, *CmAPX17*, *CmAPX24*, and *CmAPX27* are likely involved in melon’s salt stress response. The expression of *CmAPX17*, *CmAPX24*, and *CmAPX27* and the activity of their encoded APX enzymes were enhanced to different degrees under 150 mM NaCl treatment. This observation is similar to the findings in previous research revealing that plants can increase APX enzyme activity when they are faced with oxidative stress [37,38,39,40]. In addition, the expression of *CmAPX27* was higher than that of *CmAPX17* and *CmAPX24*; thus, we speculated that *CmAPX27* plays a key role in the response of melon to salt stress. Salt stress induces the accumulation of ROS in plants, and silencing of *CmAPX27* reduces the ROS-scavenging ability of melon, which confirms the antioxidant function of the protein encoded by *CmAPX27*. Under salt stress treatment with 150 mM NaCl, the leaves of *CmAPX27*-silenced plants appeared yellow and curled after 48 h; the seedlings dried up and died at around 72 h. Chlorosis was also pronounced. In contrast, only the leaf edges of NS plants were yellowed and slightly curled. The expression of *CmAPX27* and the activity of its encoded APX enzyme were significantly down-regulated and differed significantly among tissues examined in *CmAPX27*-silenced plants.

**Figure 10:**
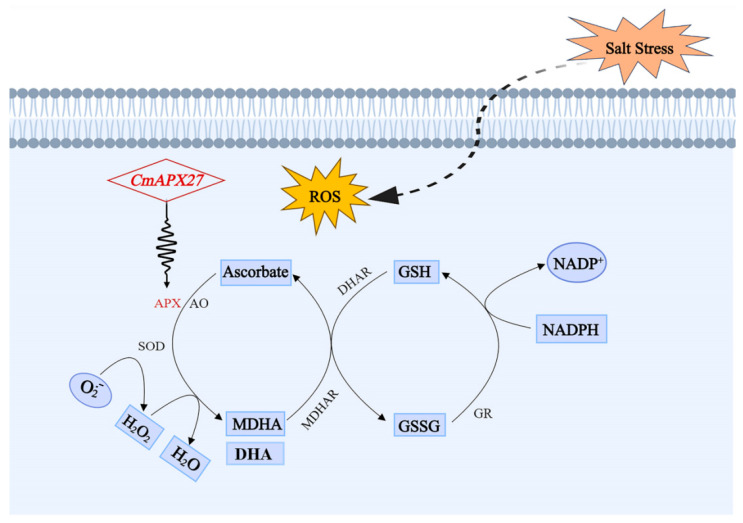
Schematic diagram of the ascorbate–glutathione pathway.

Plant antioxidant systems maintain redox and osmotic homeostasis in plants and promote their resistance to biotic and abiotic stresses [27,41,42,43,44]. Under salt stress, ROS accumulate in large quantities in plants, and excess ROS are scavenged via the ascorbate–glutathione cycle (ASC–GSH); APX is a crucial component of the ASC–GSH cycle (Figure 10) [41,42]. The scavenging of ROS via the ASC–GSH pathway induces ephemeral abundance changes in most intermediates within this pathway [41,45,46,47]. Changes in the AsA content, redox state (AsA/DHA ratio), and activities of enzymes that catalyze its synthesis and metabolism play key roles in plants’ response to salt stress. AsA and GSH are key molecules that can enhance plants’ resistance to oxidative stress; these enzymes are abundant in organelles. Maintaining a high reduced/oxidized ratio of these enzymes in plant cells, which is important for scavenging intracellular ROS, is mainly achieved via GR, MDHAR, and DHAR, using NADPH as a reducing agent. Under salt stress, the dynamic equilibrium of ROS in plants is altered, and the rate of O_2_^.−^ production and the content of H_2_O_2_ increase; as these ROS are highly toxic to cells, the rapid scavenging of H_2_O_2_ by APX is critically important. The expression of *CmAPX27* enhanced APX activity, which increased the stress resistance of plants, as well as the AsA/DHA ratio, which promoted ROS scavenging via the ASC–GSH pathway. APX activity and the AsA/DHA ratio decreased in *CmAPX27*-silenced plants under salt stress conditions, and this led to a decrease in ROS scavenging by the ASC–GSH cycle, which resulted in the accumulation of ROS, apoptosis, and, eventually, plant death. APX activity thus plays a key role in mediating the scavenging of excess ROS via the ASC–GSH pathway. *CmAPX27* might play a key role in regulating the response to salt stress in melon.

## 4. Materials and Methods

### 4.1. Identification of APX Genes in the Melon Genome

Melon *APX* genes were identified based on melon (v3.6.1) genomic information acquired from *Cucurbitaceae* (http://cucurbitgenomics.org/, accessed on 5 January 2023) using the Hidden Markov Model (HMM) and BLAST methods. First, using the TBtools software, the ascorbate peroxidase domain (PF00141) file in the HMM format was utilized as the query to search against the Pfam (http://pfam.xfam.org/, accessed on 12 January 2023) database for genes encoding APX structural domain-containing proteins [31]. In addition, the amino acid sequences of the eight APX proteins from model plants Arabidopsis and rice were acquired based on their gene sequences deposited, respectively, in the Arabidopsis genome (https://www.arabidopsis.org/, accessed on 18 January 2023) and RiceData (https://www.ricedata.cn/gene/, accessed on 18 January 2023) databases, to conduct a BLAST analysis of the melon genome to identify possible APX genes with TBtools software [48,49]. We also utilized the Uniprot database (http://www.uniprot.org/, accessed on 18 January 2023), the SMART database (http://smart.embl.de/, accessed on 18 January 2023), and the Pfam database (http://pfam.xfam.org/, accessed on 18 January 2023) to identify conserved CmAPX gene motifs and determined the subcellular localization of CmAPX proteins using CELLO v.2.5 [50].

### 4.2. Structure and Conserved Motif Analyses for the CmAPX Genes

We analyzed the protein sequences encoded by the *CmAPX* genes with the Multiple Expectation maximization for Motif Elicitation (MEME; http://meme-suite.org/, accessed on 20 January 2023) online software. These genes were also subjected to intron–exon architecture analysis with Gene Structure Display Server v2.0 (http://gsds.gao-lab.org/, accessed on 20 January 2023). Gene structure maps were made based on the results of these analyses [51].

### 4.3. Phylogenetic Relationships, Chromosomal Localization, and Collinearity Analyses of APX Genes in Melon

A phylogenetic tree for these CmAPX genes was generated by MEGA (v7.0) (http://megasoftware.net, accessed on 25 January 2023) via the neighbor-joining method (1000 bootstrap replicates) [52,53] and visualized using the iTOL website (https://itol.embl.de/, accessed on 25 January 2023). The chromosomal locations of *CmAPX* genes were determined according to melon genomic data in the GFF format, and TBtools was employed for establishing the chromosomal linkage map. The collinearity among the *APX* genes of melon, *A. thaliana*, and rice was analyzed to generate Circos maps.

### 4.4. RNA Sequencing (RNA-Seq) Analysis of CmAPX Genes

Expression profiles for the CmAPX genes were acquired from Cucurbitaceae (http://cucurbitgenomics.org/, accessed on 30 January 2023). Data on *CmAPX* gene expression patterns in plant tissues, including female flower, male flower, leaf, fruit, and root tissues, were obtained from publicly available data in the melon genome database. TBTools software was used to create heat maps for analysis of the RNA-seq data [54].

### 4.5. Plant Material and Salt Stress Treatment

Melon material X055 was obtained from the melon research group of the Horticulture Department of Northeast Agricultural University. Seeds were soaked in warm broth for 8 h to induce germination; they were then planted into nutrient pots. Salt stress treatment, NaCl solution (150 mM), was applied to melon seedlings 35 days after sowing; after making phenotypic observations, leaf, stem, and root tissues of melon seedlings were collected, frozen promptly with liquid nitrogen, and preserved in a −80 °C freezer for *CmAPX* gene expression analyses.

### 4.6. Ascorbate Peroxidase (APX) Activity Assay

APX activity was measured using an APX activity assay kit (Solarbio, Beijing, China) and an enzyme labeler according to the manufacturer’s instructions. Specifically, approximately 0.1 g of melon tissue was ground into powder in a grinder; 1 mL of extraction solution was then added for homogenization in an ice bath. This was followed by centrifugation at 13,000× *g* for 20 min at 4 °C, and measurements were made using the supernatant, which was collected on ice.

### 4.7. Cloning of APX Genes from Melon

*CmAPX* gene sequences were cloned from the melon variety X055 utilizing the following PCR program: a 5 min initial long denaturation step at 95 °C, followed by 35 rounds of amplification that involved a 30 s short denaturation step at 95 °C, a 30 s annealing step at 50 °C, and a 90 s elongation step at 72 °C. After ligating the target fragment to the pCE2 TA/Blunt-Zero vector, it was subjected to first-generation DNA sequencing by Sangon Biotech Co., Ltd. (Shanghai, China); the sequences were then compared.

### 4.8. CmAPX Gene Expression Analysis

Root, stem, and leaf samples were collected from the plants subjected to 0 h, 3 h, 6 h, 12 h, 24 h, 48 h, and 72 h of salt stress treatment with 150 mM NaCl. The TRIzol method was used to extract tissue RNA, which was subsequently reverse-transcribed into complementary DNA (cDNA) utilizing a Toyobo master mix for reverse transcription (Toyobo, Osaka, Japan). The synthesized cDNA served as the template for qRT-PCR assays using SYBR Green I as the fluorescence dye. The following program was utilized for qRT-PCR assays: a 1 min pre-denaturation step at 95 °C, followed by 35 rounds of 95 °C for 15 s, 58 °C for 20 s, and 72 °C for 15 s. The relative mRNA abundance of *CmAPX* genes was assessed by the 2^−ΔΔCt^ method, with *MELO3C023264* (Actin) serving as the internal reference gene [55,56].

### 4.9. Functional Characterization of the Candidate Gene CmAPX27

We silenced the candidate gene *CmAPX27* (*MELO3C020719.2.1*) employing the virus-induced gene-silencing (VIGS) method to determine its function. We used the pV190 vector to construct two recombinant vectors containing 300 bp *CmAPX27*-specific and 300 bp *PDS* sequences [57]; the resulting target fragments were inserted into the *Bam HI*-cleaved pV190 vector via homologous recombination, and these two vectors were referred to as pV190-CmAPX27 and pV190-PDS. The melon leaf injection method was used; phenotypic observations were made after the target genes were silenced. When symptoms of photobleaching (15 dpi) appeared on the leaves of *PDS*-silenced plants, *CmAPX27*-silenced plants were subjected to 150 mM NaCl treatment; phenotypic observations were made and root, stem, and leaf tissues treated for different periods were subjected to gene expression analysis.

### 4.10. Statistical Analysis

The statistical data of this study were expressed as the mean ± standard error (x ± s) of three biological replications. We saved our data in Microsoft Excel (2019 version) files and utilized GraphPad Prism 8.0 to perform all statistical analyses.

## 5. Conclusions

Our bioinformatics analysis revealed a total of 28 *CmAPX* genes. Analysis of the transcriptome data revealed that these 28 *CmAPX* genes were differentially expressed among tissues. These 28 *CmAPX* genes were cloned, and the amino acid sequences of the proteins encoded by these *CmAPX* genes were compared with those of the proteins encoded by three salt stress-related genes (*OsAPX2*, *AAA33645.1*, and *AGW23429.1*). Three *CmAPX* genes (*CmAPX17*, *CmAPX24*, and *CmAPX27*) in Clade I were involved in the response to salt stress in melon. Analysis of the expression patterns of these three *CmAPX* candidate genes and the activity of their encoded APX enzymes under 150 mM NaCl treatment revealed that *CmAPX27* (*MELO3C020719.2.1*) plays a key role in the response to salt stress in melon. Exposure of *CmAPX27*-silenced plants to 150 mM NaCl treatment indicated that *CmAPX27* plays a key role in regulating the response to salt stress in melon. The expression of *CmAPX* genes is closely related to APX activity.

The results of our study enhance our understanding of *APX* genes in melon, clarify the function of *CmAPX27* genes, and establish the importance of *CmAPX27* in the ASC–GSH cycle. Overall, our findings will aid future studies of this gene.

## Figures and Tables

**Table 1:** Data for 28 *CmAPX* genes identified in the melon genome.

Gene Name	Gene ID	Genomic Region	CDS Length (bp)	Exon	Intron	Protein Length (aa)	PI	Molecular Weight/kDa	GRAVY	Subcellular Localization
*CmAPX1*	*MELO3C013362.2*	chr01 (16914932, 16920143)	471	5	4	156	5.82	17.26	−0.245	Cytoplasmic
*CmAPX2*	*MELO3C017120.2*	chr02 (25825071, 25826275)	984	1	0	327	8.47	35.64	−0.084	Extracellular
*CmAPX3*	*MELO3C008186.2*	chr03 (1701410, 1704553)	1008	3	2	335	6.15	36.09	−0.124	Chloroplast
*CmAPX4*	*MELO3C010627.2*	chr03 (8704812, 8712281)	1251	12	11	416	8.45	45.04	−0.411	Chloroplast
*CmAPX5*	*MELO3C011261.2*	chr03 (27375748, 27380146)	1008	10	9	335	7.61	36.65	−0.079	Chloroplast
*CmAPX6*	*MELO3C003559.2*	chr04 (2265163, 2269072)	819	9	8	272	8.67	29.51	−0.251	Chloroplast
*CmAPX7*	*MELO3C009924.2*	chr04 (28104983, 28106495)	1107	1	0	368	8.74	41.49	−0.077	Plasma membrane
*CmAPX8*	*MELO3C014658.2*	chr05 (595378, 597928)	957	4	3	318	6.43	34.67	−0.125	Extracellular
*CmAPX9*	*MELO3C014657.2*	chr05 (603858, 605423)	1038	4	3	345	6.01	37.11	−0.182	Chloroplast
*CmAPX10*	*MELO3C014656.2*	chr05 (606283, 608027)	1038	4	3	345	5.69	37.19	−0.234	Chloroplast
*CmAPX11*	*MELO3C014655.2*	chr05 (613307, 615280)	990	3	2	329	5.92	36.67	−0.336	Extracellular
*CmAPX12*	*MELO3C014654.2*	chr05 (618721, 620283)	1002	3	2	333	8.09	36.36	−0.060	Extracellular
*CmAPX13*	*MELO3C006862.2*	chr06 (6793499, 6795861)	951	3	2	316	7.56	34.69	−0.101	Extracellular
*CmAPX14*	*MELO3C016943.2*	chr07 (948078, 951116)	984	3	2	327	5.32	34.92	0.040	Chloroplast
*CmAPX15*	*MELO3C016405.2*	chr07 (24195633, 24198118)	1032	4	3	343	9.12	37.37	−0.296	Extracellular
*CmAPX16*	*MELO3C017603.2*	chr07 (25625229, 25628495)	759	2	1	252	5.36	28.14	−0.261	Cytoplasmic
*CmAPX17*	*MELO3C007923.2*	chr08 (6265300, 6269688)	990	10	9	329	8.75	36.59	−0.242	Cytoplasmic
*CmAPX18*	*MELO3C005456.2*	chr09 (21737263, 21739391)	1014	4	3	337	4.53	35.93	−0.046	Extracellular
*CmAPX19*	*MELO3C022604.2*	chr10 (16715480, 16716883)	951	3	2	316	8.07	34.27	−0.079	Plasma membrane
*CmAPX20*	*MELO3C021914.2*	chr11 (5335837, 5338322)	1008	3	2	335	5.04	37.20	−0.140	Extracellular
*CmAPX21*	*MELO3C034836.2*	chr11 (22401880, 22403778)	975	4	3	324	8.03	35.43	−0.170	Extracellular
*CmAPX22*	*MELO3C025681.2*	chr11 (27168191, 27169996)	978	4	3	325	9.15	35.43	−0.169	Chloroplast
*CmAPX23*	*MELO3C025683.2*	chr11 (27200212, 27202002)	969	3	2	322	6.78	34.93	−0.166	Extracellular
*CmAPX24*	*MELO3C025724.2*	chr11 (27753082, 27756921)	750	9	8	249	5.86	27.63	−0.374	Cytoplasmic
*CmAPX25*	*MELO3C021259.2*	chr11 (30864215, 30866563)	993	4	3	330	4.92	35.39	0.011	Extracellular
*CmAPX26*	*MELO3C020501.2*	chr12 (349119, 351142)	960	3	2	319	5.51	34.41	−0.048	Extracellular
*CmAPX27*	*MELO3C020719.2*	chr12 (3120431, 3122973)	738	8	7	245	5.32	26.99	−0.383	Cytoplasmic
*CmAPX28*	*MELO3C002242.2*	chr12 (25369164, 25370763)	1005	2	1	334	7.59	37.07	−0.052	Extracellular

## Data Availability

All data supporting the findings of this study are included in this article.
